# Evaluation of the Coronal Malposition of the Volar Locking Plate in the Treatment of Distal Radius Fractures

**DOI:** 10.7759/cureus.26444

**Published:** 2022-06-30

**Authors:** Emirhan Usta, Ahmet O Akpolat, Ahmet N Kahraman, Adnan Kara, Yunus OC, Bekir E Kilinc

**Affiliations:** 1 Department of Orthopaedics and Traumatology, Urfa Traning and Research Hospital, Sanliurfa, TUR; 2 Department of Orthopaedics and Traumatology, Health Sciences University, Istanbul Fatih Sultan Mehmet Training and Research Hospital, Istanbul, TUR; 3 Department of Radiology, Health Sciences University, Istanbul Fatih Sultan Mehmet Training and Research Hospital, Istanbul, TUR; 4 Orthopaedics and Traumatology, Medipol Hospital, Istanbul, TUR; 5 Department of Orthopaedics and Traumatology, Beykent University School of Medicine, Istanbul, TUR; 6 Orthopaedics Surgery and Traumatology, University of Health Sciences, Fatih Sultan Mehmet Training and Research Hospital, Istanbul, TUR

**Keywords:** coronal malposition, tenosynovitis, flexor pollicis longus, volar locking plate, distal radius fracture

## Abstract

Background and objectives: Literature does not show any studies regarding plate placement problems in the coronal plane of patients with volar plating due to distal radius fracture diagnosis. We aimed to investigate the functional and laboratory results of the coronal malposition of the volar locking plate in patients with distal radius fracture treated with internal fixation.

Methods: In this retrospective study, we included patients who had volar plate fixation, were aged between 18 and 80, had no pathological fracture, had a minimum of six months of follow-up, and had the same rehabilitation protocol. We consider the angle subtended on the coronal axis between the distal radius long axis and the distal radius locking plate as coronal malposition. We named the coronal malposition angle the "AYE Angle." Patients with an AYE angle of over 1 degree were evaluated under group 1. Patients with an AYE angle of 0-1 degrees were evaluated under group 2. Radiological parameters were taken from AP-Lateral X-ray views. Superficial University System of Georgia (USG) examinations were applied to detect tendon problems. The DASH and QUICK-DASH scoring systems were used for clinical evaluation. Grip strength was measured with a dynamometer in all patients. All results were compared between the two groups.

Results: Thirteen patients were female and 27 patients were male. Nineteen patients who had coronal malposition were added to group 1, while 21 patients who had no coronal malposition were added to group 2. Fifteen patients had normal USG results in group 2, while 18 patients had edema around the flexor pollicis longus (FPL) tendon as a result of USG in group 1. Statistically, a significant difference was detected between the two groups in terms of the amount of tenosynovitis around FPL (p=0.01).

A statistically significant relationship was found between USG grading and malposition grading. The study revealed that a higher rate of USG grade 2 was found in patients with malposition grade 2 (90.9%), while a higher rate of USG grade 1 (50%) was observed in patients with malposition grade 1 (p=0.01).

A statistically significant difference was not found between Soong grading and USG in terms of the level of tenosynovitis around the FPL tendon. The amount of tenosynovitis detected around the FPL tendon was 62.5% for Soong and grade 0 level, 60.7% for grade 1 level, and 50% for grade 2 level. There was no statistically significant difference between the two groups in the DASH and QUICK-DASH scoring systems (p=0.96). There was no statistically significant difference between the two groups in the grip strength (p=0.52).

Conclusion: Coronal plate position in the treatment of the distal radius fracture is important to avoid potential flexor tendon problems. The volar plate position should be adjusted properly both in the coronal and sagittal axes.

## Introduction

Distal radius fractures are one of the most common types of bone fractures [1]. Nearly 18-22% of all emergency department visits for fracture incidents and 75-80% of all forearm fractures consist of distal radius fractures [2-3]. Today, volar plate fixation and internal fixation are the most popular methods used to treat distal radius fracture as they provide advantages such as ensuring full anatomic reduction and early start of mobility [4]. Tendon problems, nerve disorders, loss of reduction-fixation, nonunion, infection, arthrosis, complex regional pain syndrome, Dupuytren’s disease, and compartment syndrome can be regarded as the most common complications that may occur as a result of treatment of distal radius fracture [5]. As for tendon complications, problems may occur in both flexor and extensor tendons [6].

The literature does not show any studies regarding plate placement problems in the coronal plane of patients who underwent volar plating due to a diagnosis of distal radius fracture. The study that addresses malposition in the sagittal plane investigates tendon problems and provides limited data as it focuses on a single plane [7].

In line with our hypothesis, we argue that the angle subtended between volar plate placement in the coronal plane and the radius shaft can be tolerated up to a certain degree and that an increasing angle of coronal malposition will have a negative effect on clinical, functional, and laboratory results. We consider that the tendon problem will not be related only to plate placement in the sagittal plane, but plate placement in the coronal plane must also be evaluated simultaneously.

This study aims to investigate the functional and laboratory results of the coronal malposition of the volar locking plate in patients with distal radius fracture treated with internal fixation.

## Materials and methods

Our study was conducted at a single institution with the registration number FSMEAH-KAEK 2020/79. Our research was conducted in line with the Good Clinical Practice Guidelines, which is the last text of the Declaration of Helsinki. Forty-seven patients who had distal radius fractures between March 2019 and July 2020 and who were treated with open reduction and internal fixation by the use of a volar locking plates were evaluated retrospectively.

Our inclusion criteria were patients who were treated with a 3.5 mm volar plate (TST firm) with a 25 mm distal part, aged over 18 and below 80 years, without any pathological fracture, no neurological deficit of the upper extremity, with at least a six month follow-up period, without any different but simultaneous fractures of the same extremity, and who had the same postoperative rehabilitation program. Seven patients were excluded from the study. In total, 40 patients who met the study criteria were included.

All patients had pronator quadratus repair.

**Figure 1:**
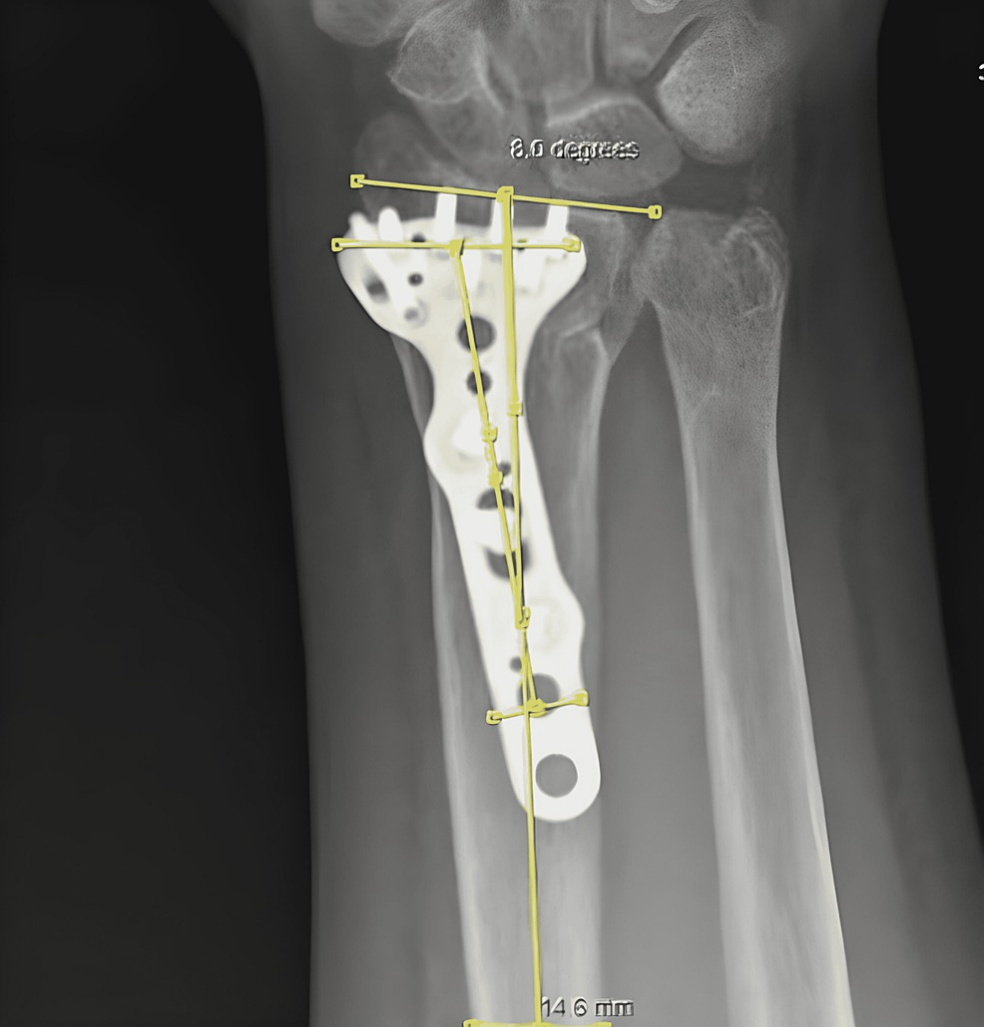
Coronal malposition measurement A patient with AYE angle of 8 degrees

**Figure 2:**
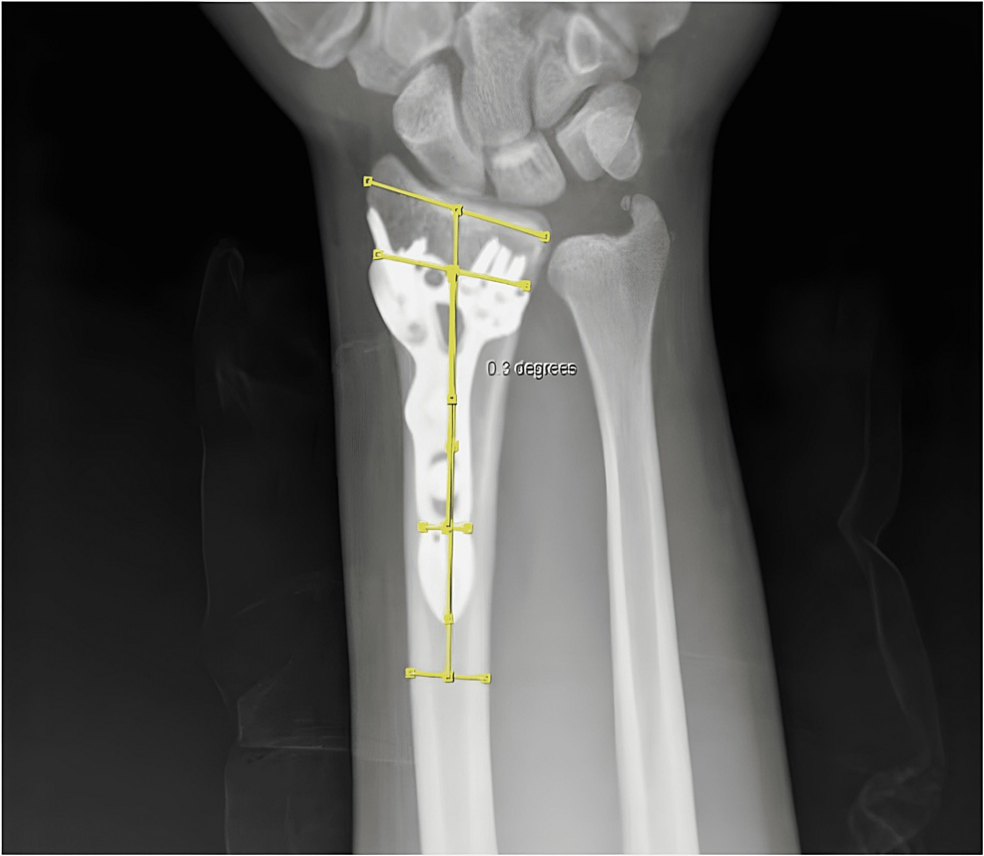
Coronal malposition measurement A patient with AYE angle of 0.3 degrees

We consider the angle subtended on the coronal axis between the distal radius long axis and the distal radius locking plate as coronal malposition. We named the coronal malposition angle that we put forward in our study the "AYE Angle." AYE angle can be described as the angle between the line that passes parallel to the long axis of the distal radius and through its midline and the line that passes parallel to the long axis of the plate and its midline (Figures 1-2). Patients with an AYE angle of over 1 degree were evaluated under group 1. Patients with an AYE angle of 0-1 degrees were evaluated under group 2.

Coronal malposition angles were measured by two different physicians. Inter-observer and intra-observer reliability scores were calculated based on the measurements. Kappa statistical measure was used to find reliability index of the study. During postoperative follow-up, wrist AP-Lateral X-ray and superficial USG examinations were applied. The DASH and QUICK-DASH scoring systems were used for clinical evaluation. Radiological evaluations were conducted in collaboration with a 20-year musculoskeletal experienced radiologist who was not informed about the clinical results and patients’ medical histories. Soong grade 7 and AO fracture types of patients were also detected. The amount of tenosynovitis around flexor pollicis longus (FPL) was evaluated through USG.

We created a 3-grade system for our evaluation depending on whether patients in the study group have coronal malposition or not: patients with coronal malposition or AYE angle between 0 and 1 degree were categorized under grade 0. Patients with an AYE angle of between 1 and 5 degrees under grade 1. Patients with an AYE angle that is higher than 5 degrees under grade 2.

Superficial USG was applied by the use of the Philips EPIQ Elite Color Doppler ultrasonography device in our clinic for both the experimental group and the control group. An 8-14 Mhz high-resolution linear transducer was used for the examination. The FPL tendon, plate and screws, and distal radius were evaluated through ultrasonography when the patient’s hand was in a supine position and slightly extended.

**Figure 3:**
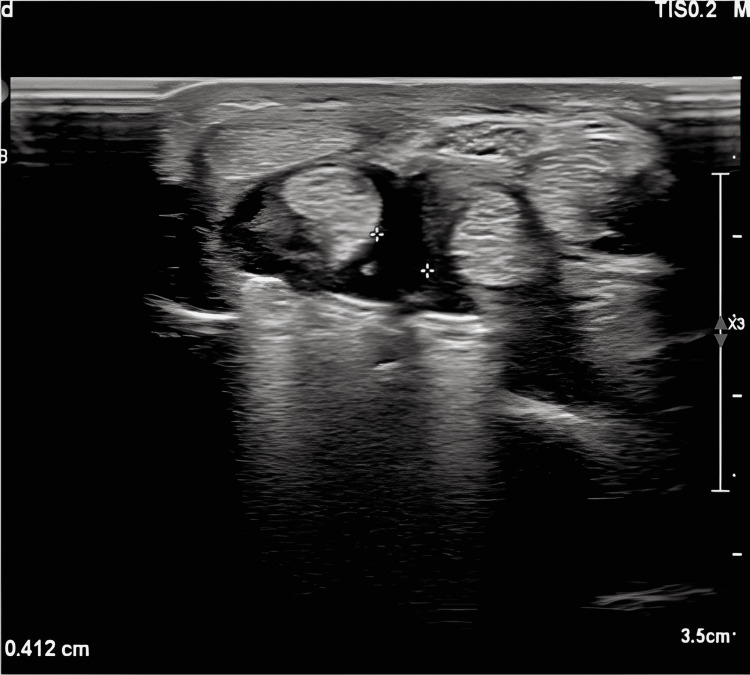
A patient from group 1 with 4-mm tenosynovitis around the flexor pollicis longus tendon

We categorized the fluid around FPL into three grades (Figure 3). Grade 0: Fluid was not detected around the FPL tendon. Grade 1: Fluid of 0-2 mm was detected around the FPL tendon. Grade 2: Fluid that is over 2 mm was detected around the FPL tendon.

In our study, we investigated the relationship between coronal malposition and flexor tendinopathies by using these grading systems. Grip strength was measured with a dynamometer in all patients (Jamar; Therapeutic Equipment Corp., Clifton, NJ, USA). All results were compared statistically between the two groups.

Statistical analysis

The descriptive statistics of the study were taken as mean, standard deviation, percentage, and frequency. The Mann-Whitney U was conducted to examine differences between patient groups in terms of score, age, amount of edema, and follow-up periods. A chi-square test was used to determine differences with regard to USG grades in accordance with patient groups. For the analyses, the critical decision value was taken as 0.05. Results were evaluated within a confidence interval of 90% and statistical significance was described as p˂0.05. The SPSS 25.00 package program (IBM SPSS, Armonk, NY) was used to end the analysis. The power level and impact magnitude were calculated by the use of G*Power version 3.1.7.

## Results

Thirteen patients were female and 27 patients were male. Ten patients had left wrist surgery, while 30 had right wrist surgery. All of the patients were declared to be right-handed.

Nineteen patients who had coronal malposition were added to group 1, while 21 patients who had no coronal malposition were added to group 2. The mean volar tilt angle of group 1 was 10.8 ± 2.1, while that of group 2 was 11.2 ± 2.4. There was no statistically significant difference between the two groups (p=0.96). The mean radial inclination of group 1 was 21.86 ± 2.50, while that of group 2 was 22.03 ± 2.29. There was no statistically significant difference between the two groups (p=0.524). The follow-up periods of both groups were 24.11 ± 11.21 and 23.9 ± 13.28 months, respectively. There was no statistically significant difference between the two groups (p=0.96). Fifteen patients had normal University System of Georgia (USG) results in group 2, while 18 patients had edema around the FPL tendon as a result of USG in group 1. A statistically significant difference was detected between the two groups in terms of the amount of tenosynovitis around FPL (p=0.01) (Table 1).

**Table 1:** USG results based on study groups *Significant difference of 0.05 USG: University System of Georgia

USG		Group		p-Value
		Group 1	Group 2	
Normal	n	1	15	0.01*
	%	6.3%	93.8%	
With edema	n	18	6	
	%	75.0%	25.0%	

There is a significant difference between the two groups in terms of USG grades. It was found that 93.8% of patients with USG grade 0 were included in group 2, while 62.5% of grade 1 patients and 81.3% of grade 2 patients were in group 1 (Table 2) (p=0.01).

**Table 2:** USG grades based on two groups *Significant difference of 0.05 USG: University System of Georgia

USG Grade		Group		p-Value
		Group 1	Group 2	
Grade 0	n	1	15	0.01*
	%	6.3%	93.8%	
Grade 1	n	5	3	
	%	62.5%	37.5%	
Grade 2	n	13	3	
	%	81.3%	18.8%	

The study revealed that USG grades changed in group 1 depending on AYE angles. Patients with USG grade 2 had higher AYE angles (p=0.01). A statistically significant relationship was found between the increase in AYE angle and USG grade rise in group 1 (Table 3).

**Table 3:** USG grade and AYE angle *Significant difference of 0.05 USG: University System of Georgia

Grade	X	s.s	p-Value
1	3.21	0.91	0.01*
2	7.37	1.81	

A statistically significant relationship was found between USG grading and malposition grading. The study revealed that a higher rate of USG grade 2 was found in patients with malposition grade 2 (90.9%), while a higher rate of USG grade 1 (50%) was observed in patients with malposition grade 1 (Table 4) (p=0.03).

**Table 4:** USG grade and malposition grade *Significant difference of 0.05 USG: University System of Georgia

Malposition grade		USG grade			p-Value
		Grade 0	Grade 1	Grade 2	
1	n	1	4	3	0.03*
	%	12.5%	50.0%	37.5%	
2	n	0	1	10	
	%	0.0%	9.1%	90.9%	

A statistically significant difference was not found between Soong grading and USG in terms of the level of tenosynovitis around the FPL tendon. The amount of tenosynovitis detected around the FPL tendon was 62.5% for Soong and grade 0 level, 60.7% for grade 1 level, and 50% for grade 2 level (Table 5) (p=0.91).

**Table 5:** Soong grade and USG results USG: University System of Georgia

SOONG grade		USG		p-Value
		Normal	With tenosynovitis	
Grade 0	n	3	5	0.91
	%	37.5%	62.5%	
Grade 1	n	11	17	
	%	39.3%	60.7%	
Grade 2	n	2	2	
	%	50.0%	50.0%	

A statistically significant difference was not found between the Soong grading system and USG grading. It can be stated that Soong grading and USG comply with one another by 37.5% for grade 0 level, 21.4% for grade 1 level, and 25% for grade 2 level (Table 6) (p=0.93).

**Table 6:** Soong grading and USG grading USG: University System of Georgia

SOONG grade		USG grade			p-Value
		Grade 0	Grade 1	Grade 2	
Grade 0	n	3	1	4	0.93
	%	37.5%	12.5%	50.0%	
Grade 1	n	11	6	11	
	%	39.3%	21.4%	39.3%	
Grade 2	n	2	1	1	
	%	50.0%	25.0%	25.0%	

There was no statistically significant difference between the two groups in the DASH and QUICK-DASH scoring systems (p=0.96). There was no statistically significant difference between the two groups in the grip strength (p=0.52).

## Discussion

Our study reveals that there is a statistically significant relationship between coronal positioning of the volar plate and flexor tendon problems. We determined that as the AYE angle increases, the incidence of FPL tenosynovitis rises. This study investigates the effect of plate placement in the coronal plane on the FPL tendon and the amount of tenosynovitis through USG examination. The literature does not show any studies which are related to a malposition of the volar plate in the coronal plane for the treatment of distal radius fractures.

Distal radius fractures, which can be considered simple and stable today, are treated mostly by the use of conservative methods. Operative treatment is preferred mostly for unstable and intra-articular fractures. The main purpose of surgery is to promote a rapid return to function while minimizing the risk of late arthritis by means of accurate articular reduction and stabilization [8].

Many patients may present with a complaint of tenosynovitis following internal fixation with a volar plate for distal radius fractures [9]. Our study investigates the effect of coronal malposition on the development of tenosynovitis and determines the amount of tenosynovitis around the FPL tendon through superficial ultrasonography.

USG’s peculiar advantages, such as cost-effectiveness, accessibility, relatively short analysis time, and the opportunity to conduct dynamic and real-time comparisons with the contralateral side, make it easier to use for musculoskeletal examinations [9-12]. One study reported on the amount of tendosynovitis found on USG, related to symptomatic and asymptomatic tendon issues after plate implantation [13]. Another study scored the amount of tenosynovitis with USG in patients with rheumatoid arthritis [14]. Another study reported that the extensor tendon complications and dorsal screw penetrations were detected through USG examination in patients who were treated with internal fixation with a volar plate. It has been reported in the literature that USG can be used to determine and score tendon complications. USG has also been used to investigate flexor tendinopathies in patients treated with volar plate fixation of distal radius fractures [13-16]. We studied the FPL tendon in particular, since it is the closest tendon to bone and, as it is the closest tendon to bone and, therefore, the closest tendon to the volar plate [13,15,16].

In our study, a statistical difference was detected, through USG grading, between the two groups in terms of the amount of tenosynovitis around the FPL tendon. It was observed that the rate of USG grades changes depending on group 1. It was revealed that the great majority of patients with USG grade 0 were included in group 2, while the great majority of patients with grades 1 and 2 consisted of patients from group 1. AYE angle and USG grading were found to be statistically correlated.

It was detected that as the AYE angle increases, a statistically significant rise occurs in the amount of tenosynovitis measured through USG and in grading. However, some studies suggest that complications may occur due to the placement of the plate on the sagittal axis [7]. Soong et al. expressed in their study that the risk of flexor tendinopathies and rupture increases as the volar locking plate is distal to the volar rim or volar to watershed. They reported a three-grade classification in their studies. Their study showed that as grade increases, the prevalence of flexor tendinopathy also increases [7]. Our study did not reveal any statistically significant relationship between the Soong grading system and USG grading. In the literature, the study conducted by DeGeorge et al. in 2020 caught our attention. According to the study conducted by DeGeorge et al., Soong classification could not determine flexor tendinopathies and tendon ruptures. Also in our study, Soong classification did not provide statistically significant data in terms of determining flexor tenosynovitis through USG. Our data supports the study of DeGeorge et al. [17]. In our study, we established that plate placement in the sagittal plane is not compatible with the criteria set by Soong et al. Our study did not reveal any statistically significant results as to tenosynovitis in the sagittal plate position that is expected to cause absolute tenosynovitis according to Soong criteria. We concluded that Soong evaluation criteria are more reliable when the "AYE" angle, which is described in our study, is incorporated into the investigation and that the incidence of tenosynovitis is statistically more significant.

Plate placement in coronal malposition for distal radius fractures may cause tenosynovitis and tendon complications. The lack of studies investigated in the literature that investigate tendon problems that may be caused by coronal plate placement renders our study valuable. The studies which investigated the relationship between flexor tendon problems and plate placement in the sagittal plane provide limited information. Our study shows that flexor tendon problems that occur after treatment of distal radius fractures with volar locking plates are not only related to positioning in the sagittal plane but also in the coronal plane.

## Conclusions

Our study shows that, for distal radius fractures, volar plate placement does have an effect on flexor tendons. We found a statistically significant correlation between the coronal plane positioning of the plate and flexor tendon problems as determined by ultrasound examination. We suggest that reliable plate placement can be achieved in the sagittal plane in line with Soong criteria and in the coronal plane by taking the "AYE" angle into consideration. In order to avoid flexor tendon complications, it is important that suitable plate positions be determined in both the coronal and sagittal planes by a thorough assessment of the fracture pattern if volar plating is considered.
